# Supplementary material for: Suicidal ideation and associated factors among pregnant women attending antenatal care at public hospitals of Harari regional state, eastern Ethiopia: A cross-sectional study
Source: PLoS One. 2024 Mar 28;19(3):e0300417. doi: 10.1371/journal.pone.0300417 (PMC10977762; doi:10.1371/journal.pone.0300417)
Supplement: S1 Dataset — (DOCX) [file pone.0300417.s001.docx]

## Code ------------

## 7.2. Participant information sheet and Consent form

First of all, we would like to thank for being willing to spend time with us. Our name is Hiwot Lemma, Kalkidan Tadesse amd Kebegna Jemal and we are a 4^th^ year undergraduate psychiatric nursing student at Haramaya university, college of medicine and health science, investigating on “Prevalence of suicidal behavior and associated factor among pregnant women attending antenatal care in public hospitals of Harari regional state”.

**Purpose of the study;** The purpose of this study is to determine the prevalence and its associated factors of suicidal behavior among pregnant women attending antenatal care in this public hospital and the study will be conducted for partial fulfillment of the requirement of the Degree of Bachelor of Science in Psychiatric nursing.

**Procedure and duration;** We are going to ask you to read and fill the questionnaire which it takes from 20-30 minutes of your time. Your willingness to fill the questionnaire will help us to identify the prevalence and associated factors of suicidal behavior among pregnant women attending antenatal care services in public hospital.

**Confidentiality:** All information collected on questionnaires will be entered into computer with special identification number. The questionnaires will be handled with greater secrecy in order to maintain confidentiality.

**Rights, Risks and Benefits:** The participation is voluntary and you are not obliged to participate without your will. You have the right to participate and withdraw from participation at any time. Refusal will not benefit or cost you anything. There is no risk associated with participation in our study. Taking part in our study is completely a voluntary choice and you have full right to refuse from participating in our study.If you agree to take part in this study, there will be no direct benefits to you, but your participation is likely help us to meet the research objective.

**Declaration of consent;** I have read the participant information sheet and I have clearly understood the purpose of the research, the risk and benefit, and issue of confidentiality. I have been given the opportunity to ask a question for things that may have been unclear. I am also informed that I have the right to withdraw from the study at any time. Therefore, I declare my voluntary consent to participate in this study with my signature as indicated below:

**Signature of participant;** ____________________ Date _________________

**Name and sign of data collector;** __________________ Date _________________

**PART 1: SOCIO DEMOGRAPHIC INFORMATION**

**Instruction:** These questions are focused on your background information.

| **No** | **Demographic information** | **Alternative response** | | | | | |
| --- | --- | --- | --- | --- | --- | --- | --- |
| SD-101 | How old are you? | Age in year ___________ | | | | | |
| SD-102 | What is your religion? | 1.Orthodox | 2.Muslim | | 3.Protestant | | |
|  |  | 4.Catholic | 5.Others__________ | | | | |
| SD-103 | What is your marital status? | 1. Single | | 2.Married | | | 3.Divorced |
|  |  | 4.Widowed | | | | | |
| SD-104 | What is the level of your education? | 1. Non-educated | | 2. 1-8 grade | | 3. 9-10grade | |
|  |  | 4.11-12 grade | | 5. Diploma | | 6.Degree and above | |
| SDQ-105 | What is your occupation? | 1. Government employed | | 1. Merchant | | 1. Farmer | |
|  |  | 1. Student | | 1. Day laborer | | 1. housewife | |
|  |  |  |  |  |  | 7.Others_________ | |
| SDQ-106 | From where is your residency? | 1. Rural 2. Urban | | | | | |
| SDQ-107 | What is your average monthly income? | _____________birr | | | | | |

**PART 2: OBSTETRICS FACTORS INFORMATIONS**

| **No.** | **Questions** | **Alternative response** | |
| --- | --- | --- | --- |
| OQ-201 | Gestation in the week | ______________weeks | |
| OQ-202 | Number parity | _________ | |
| OQ-203 | Have you ever had an abortion? | 1. No | 1. Yes |
| OQ-204 | If your response is yes for No OQ-203, the Number of abortions you experienced? | ______________ | |
| OQ-205 | Do you have an abortion intention in the current pregnancy? | 1. No | 1. Yes |
| OQ-206 | Is your pregnancy planned? | 1. No | 1. Yes |
| OQ- 207 | Do you experienced nausea or vomiting? | 1. No | 1. Yes |
| OQ-208 | Card review lab investigation urine analysis **ketone result** | 1. Negative | 1. Positive |
| OQ-209 | Card review lab investigation urine analysis **protouria result** | 0. Negative | 1. Positive |
| OQ-210 | Card review lab investigation LFT SGOT | 1. Negative | 1. Positive |
|  | ***SGPT*** | 0. Negative | 1. Positive |
| OQ-211 | Card review lab investigation TSH, | 1. Negative | 1. Positive |
|  | Card review lab investigation T3 | 0. Negative | 1. Positive |
|  | Card review lab investigation T4 | 1. Negative | 1. Positive |

**PART 3: CLINICAL FACTORS QUESTIONS (from Card review)**

| **No**. | **Chronic illness questions** | | **Alternative response** | | |  |
| --- | --- | --- | --- | --- | --- | --- |
|  |  |  | No | | Yes |  |
| CQ-301 | Do you have a known history of mental illness? | | 0 | | 1 |  |
| CQ-302 | Do any of your relatives suffer from mental illness? | | 0 | | 1 |  |
| CQ-303 | Do any of your relatives have a history of suicidal attempts? | | 0 | | 1 |  |
| CQ-304 | Do you ever have a chronic medical illness? | | 0 | | 1 |  |
| CQ-305 | If yes to C-4, what types of illness do you have? | 1. HIV/AIDS 2. Asthma 3. DM | | 1. HTN 2. Epilepsy 3. Other­­­­­ specify _________ | |  |

**PART IV- Question to Assess Depression, Anxiety, and Stress**

For each statement below, please circle the number in the column that best represents how you have been feeling in the last few weeks. Choice and circle the numbers from the given alternative as to how much it occurs on you. 0 for Did did not apply to me at all, 1for Applied to me to some degree or some of the time, 2 for Applied to me a considerable degree or a good part of the time, and 3 for Applied to me very much or most of the time.

| No. | \| Statement \| \| --- \| | Response |
| --- | --- | --- | --- |
| 401 | \| I couldn't seem to experience any positive feeling at all \| \| --- \| | 0 1 2 3 |
| 402 | \| I found it difficult to work up the initiative to do things \| \| --- \| | 0 1 2 3 |
| 403 | I felt that I had nothing to look forward to | 0 1 2 3 |
| 404 | \| I felt down-hearted and blue \| \| --- \| | 0 1 2 3 |
| 405 | \| I was unable to become enthusiastic about anything. \| \| --- \| | 0 1 2 3 |
| 406 | \| I felt I wasn't worth much as a person \| \| --- \| | 0 1 2 3 |
| 407 | \| I felt that life was meaningless \| \| --- \| | 0 1 2 3 |
| 408 | \| I was aware of the dryness of my mouth \| \| --- \| | 0 1 2 3 |
| 409 | \| I experienced breathing difficulty (eg, excessively rapid breathing, breathlessness in the absence of physical exertion) \| \| --- \| | 0 1 2 3 |
| 410 | \| I experienced trembling (eg, in the hands) \| \| --- \| | 0 1 2 3 |
| 411 | \| I was worried about situations in which I might panic and make a fool of myself \| \| --- \| | 0 1 2 3 |
| 412 | \| I felt I was close to panic \| \| --- \| | 0 1 2 3 |
| 413 | \| I was aware of the action of my heart in the absence of physical exertion (eg, sense of heart rate increase, heart missing a beat) \| \| --- \| | 0 1 2 3 |
| 414 | I felt scared without any good reason. | 0 1 2 3 |
| 415 | I found it hard to wind down | 0 1 2 3 |
| 416 | \| I tended to over-react to situations \| \| --- \| | 0 1 2 3 |
| 417 | \| I felt that I was using a lot of nervous energy \| \| --- \| | 0 1 2 3 |
| 418 | \| I found myself getting agitated \| \| --- \| | 0 1 2 3 |
| 419 | \| I found it difficult to relax \| \| --- \| | 0 1 2 3 |
| 420 | \| I was intolerant of anything that kept me from getting on with what I was doing \| \| --- \| | 0 1 2 3 |
| 421 | \| felt that I was rather touchy \| \| --- \| | 0 1 2 3 |

**PART 5: SUBSTANCE USE QUESTIONNAIRE**

| **S. No** | **Questions** | **Options** | |
| --- | --- | --- | --- |
| PS01 | Have you ever had history of substance use? | 0. Yes 1. No | |
| PS02 | If yes, which substance you use? 1. Khat 2. Cigarette 3. Alcohol 4. Hashish 5. Others (specify…… | | |
| PS03 | Do you use substance with the last 3 months? | | 0. Yes 1. No |
| PS04 | If yes, which substance you use? 1. Khat 2. Cigarette 3. Alcohol 4. Hashish 5. Others (specify…… | | |

**PART 6. SOCIAL SUPPORT QUESTIONNAIRE (OSSS-3**)

**Instruction;** For each of the following statement, please select one alternative answer, which shows how you feel about the support you have right now

| No, | Social support questionnaires | Alternative response |
| --- | --- | --- |
| 1 | How many people are so close to you that you can count on them if you have serious personal problems (choose one option)? | 1.None  2.1 or 2  3.3-5  4.More than 5 |
| 2 | How much concern do teachers and others show in what you are doing (choose one option)? | 1. No concern and interest  2.Little concern and interest  3. Uncertain  4. some  5. a lot |
| 3 | How easy is it to get practical help from friends or neighbors if you should need it (choose one option)? | 1. Very difficult  2. Difficult  3. Possible  4. Easy  5.Very easy |

**PART 7: The Pittsburgh Sleep Quality Index (PSQI)**

**Instructions:** The following questions relate to your usual sleep habits during the **past month** only. Your answers should indicate the most accurate reply for the majority of days and nights in the past month. Please answer all questions. During the past month

| **No**. | **Questions** | **Possible response** | | | | |
| --- | --- | --- | --- | --- | --- | --- |
| PSQI-1021 | When have you usually gone to bed at night? | Bed time________________ | | | | |
| PSQI-1022 | How long (in minutes) has it taken you to fall asleep each night? | Number of minutes______________ | | | | |
| PSQI-1023 | When have you usually get up in the morning? | Getting up time______________ | | | | |
| PSQI-1024 | How many hours of actual sleep do you get at night? (This may be different than the number of hours you spend in bed) | Houses of sleep per night______________ | | | | |
| PSQI-1025 | During the past month, how often have you had trouble sleeping because of you….?  Choose the following | **Not during the past**  **month (0)** | **Less than**  **Once a week (1)** | **Once or twice a**  **week (2)** | | **Three or**  **more times**  **week (3)** |
| PSQI-1025A | Cannot get to sleep within 30 minutes |  |  | |  |  |
| PSQI-1025B | Wake up in the middle of the night or early morning |  |  | |  |  |
| PSQI-1025C | Have to get up to use the bathroom |  |  | |  |  |
| PSQI-1025D | Cannot breathe comfortably |  |  | |  |  |
| PSQI-1025E | Cough or snore loudly |  |  | |  |  |
| PSQI-1025F | Feel too cold |  |  | |  |  |
| PSQI-1025G | Feel too hot |  |  | |  |  |
| PSQI-1025H | Have bad dreams |  |  | |  |  |
| PSQI-1025I | Have pain |  |  | |  |  |
| PSQI-1025J | Other reason(s), please describe, including how often you have had trouble sleeping because of this reason(s):  __________________________________ |  |  | |  |  |
| PSQI-1026 | During the past month, how often have you taken a medicine (prescribed or “over the counter”) to help you sleep? |  |  | |  |  |
| PSQI-1027 | During the past month, how often have you had trouble staying awake while driving, eating meals, or engaging in the social activity? |  |  | |  |  |
| PSQI-1028 | During the past month, how much of a problem has it been for you to keep up the enthusiasm to get things done? |  |  | |  |  |
|  | | **Very good(0)** | **Fairly good(1)** | | **Fairly bad(2)** | **Very bad(3)** |
| PSQI-1029 | During the past month, how would you rate your sleep quality overall? |  |  | |  |  |

**PART 8. Suicidal ideation and attempt questionnaires**

The following questionnaire consists of 11 items. Please read each item carefully and then pick out the one choice in each group that best describes past suicidal ideation, plan, attempt including today. Be sure that you do not choose more than one statement for any item.

| **No**. | **Questionnaires(CIDI)** | | **possible response** | |
| --- | --- | --- | --- | --- |
| SQ-1031 | Have you ever seriously thought about committing suicide? | | 1. No | 1. Yes |
| SQ-1032 | Have you seriously thought about committing suicide within the last 1 month? | | 1. No | 1. Yes |
| SQ-1033 | Have you ever made a plan to commit suicide? | | 1. No | 1. Yes |
| SQ-1034 | Have you ever attempt suicide? | | 1. No | 1. Yes |
| SQ-1035 | If yes to SQ-1036 how many times? | | 1. Once 2. Twice 3. More than twice | |
| SQ-1036 | Have you attempted committing suicide in the last 1 month? | | 1. No 2. Yes | |
| SQ-1037 | If yes to SQ-1036 or SQ-1038 what methods did  You use to commit suicide? | 1.Hanging   1. Poisoning 2. Use sharp tools | 1. Jumping from a high place 2. Other specify------------ | |
| SQ-1038 | Which one of the following responses most describes your suicide attempt? | 1. I made a serious attempt to kill myself and it was only luck that I did not succeed  2. I tried to kill myself, but knew that the method was not fool-proof  3. My attempt was to cry for help. I did not intended to die  4.other specify_________________ | | |
| SQ-1039 | Can you tell the reason(s) for the attempt? | 1. Family conflict 2. Death in family 3. Financial loss | 1. Mental illness 2. Physical illness 3. Others specify ____ | |

**PART 9: INTIMATE PARTNER VIOLENCE QUESTIONNAIRE (AAS)**

| No. | Intimate partner violence questionnaires | Alternative response |
| --- | --- | --- |
|  | Have you ever been emotionally or physically abused by your partner or someone important to you? | 1. No 2. Yes |
|  | Within the last year, have you been hit, slapped, kicked or otherwise physically hurt by someone? | 1. No 2. Yes |
| 3. | Since you have been pregnant, have you been hit, slapped, kicked or otherwise physically hurt by someone? | 1. No 2. Yes |
| 4. | Within the past year, has anyone forced you to have sexual activities? | 1. No 2. Yes |
