# Supplementary material for: Suicidal ideation and associated factors among pregnant women attending antenatal care at public hospitals of Harari regional state, eastern Ethiopia: A cross-sectional study
Source: PLoS One. 2024 Mar 28;19(3):e0300417. doi: 10.1371/journal.pone.0300417 (PMC10977762; doi:10.1371/journal.pone.0300417)
Supplement: Attachment — Submitted filename: Rebuttal letter.docx [file pone.0300417.s004.docx]

**REBUTTAL LETTER**

We were pleased to have an opportunity to revise our manuscript entitled “**Suicidal ideation and associated factor among pregnant women in Eastern part of Ethiopia**”. In the revised manuscript, we have carefully considered journal requirements, the editor's and reviewers suggestions and comments and we have tried to address them accordingly. The editor’s and reviewer’s comments were very helpful overall, and we are appreciative of such constructive feedback on our original submission. After addressing the issues raised, we feel the quality of the paper is much improved. The second comment of the editor is responded to at the end of the first comment of the editor.

Sincerely,

On behalf of all authors,

Tilahun Bete

Please include the following items when submitting your revised manuscript:

- A rebuttal letter that responds to each point raised by the academic editor and reviewer(s). You should upload this letter as a separate file labeled 'Response to Reviewers'.
- A marked-up copy of your manuscript that highlights changes made to the original version. You should upload this as a separate file labeled 'Revised Manuscript with Track Changes'.
- An unmarked version of your revised paper without tracked changes. You should upload this as a separate file labeled 'Manuscript'.

Journal requirements:

When submitting your revision, we need you to address these additional requirements.

1. Please ensure that your manuscript meets PLOS ONE's style requirements, including those for file naming. The PLOS ONE style templates can be found at

[https://journals.plos.org/plosone/s/file?id=wjVg/PLOSOne_formatting_sample_main_body.pdf](https://journals.plos.org/plosone/s/file?id=wjVg/PLOSOne_formatting_sample_main_body.pdf#inbox/_blank) and

[https://journals.plos.org/plosone/s/file?id=ba62/PLOSOne_formatting_sample_title_authors_affiliations.pdf](https://journals.plos.org/plosone/s/file?id=ba62/PLOSOne_formatting_sample_title_authors_affiliations.pdf#inbox/_blank)

***Author’s response: We have checked the templates and made the adjustments to meet the journal requirements. Some of them are:***

- ***We corrected all major sections (Abstract, Introduction, Materials and Methods, Results, Discussion) to level 1 heading, bold type, 18pt font, and sentence case***
- ***We corrected sub-sections of major sections to Level 2 heading, bold type, 16pt font, and sentence case.***
- ***We corrected sub-sections of within level 2 headings to level 3 heading, bold type, 14pt font, and sentence case.***
- ***We used appropriate file naming.***

***We hope that it now fits the style requirements. Thank you***

2. Thank you for stating the following in the Funding Section of your manuscript:

“The whole required (material and humanitarian) cost for this research work was covered by Haramaya University.”

We note that you have provided funding information that is not currently declared in your Funding Statement. However, funding information should not appear in the Acknowledgments section or other areas of your manuscript. We will only publish funding information present in the Funding Statement section of the online submission form.

Please remove any funding-related text from the manuscript and let us know how you would like to update your Funding Statement. Currently, your Funding Statement reads as follows:

“The author(s) received no specific funding for this work.”

Please include your amended statements within your cover letter; we will change the online submission form on your behalf.

***Authors’ response: We have accepted the comment and we removed any funding-related text from the acknowledgments section or other areas of the manuscript in the revised version. We do not want to change the funding statement stated on the online submission form since it is similar with what we have stated in the funding section. Thank you.***

3. In the online submission form, you indicated that [The datasets used and/or analyzed during the current study are available from the corresponding author upon reasonable request.]

All PLOS journals now require all data underlying the findings described in their manuscript to be freely available to other researchers, either 1. In a public repository, 2. Within the manuscript itself, or 3. Uploaded as supplementary information.

This policy applies to all data except where public deposition would breach compliance with the protocol approved by your research ethics board. If your data cannot be made publicly available for ethical or legal reasons (e.g., public availability would compromise patient privacy), please explain your reasons on resubmission and your exemption request will be escalated for approval.

***Authors’ response: We have accepted the comment and we have uploaded all the data underlying the findings described in the manuscript as supplementary information. Then, we have changed the statement in Availability of data and materials section as “All relevant data are within the paper and it’s supporting information files” in the revised version of the manuscript. Thank you.***

4. Your ethics statement should only appear in the Methods section of your manuscript. If your ethics statement is written in any section besides the Methods, please move it to the Methods section and delete it from any other section. Please ensure that your ethics statement is included in your manuscript, as the ethics statement entered into the online submission form will not be published alongside your manuscript.

***Authors’ response: We have accepted the comment and we moved it to the Methods section and deleted it from any other section. Thank you.***

Additional Editor Comments:

Dear editor , I would like to thank you for writing a good paper.
But I do have some concerns and corrections
1. Have you done any laboratory experiments? for a statement, " those mothers with hyperemesis gravidarum secondary to other medical conditions such as thyroid
diseases and liver diseases were excluded from the study after proven investigation." If so, please state the data collection tool.

***Authors’ response: Dear editor, thank you for your concern. In order to decrease the potential confounders, we did the lab investigations for those participants suspected for underlying medical conditions like thyroid disease (sent for T3, T4, and TSH) and liver disease (sent for liver function test like SGOT, SGPT). We included this in the data collection tool section in the revised manuscript. Thank you.***

2. Please rewrite a reliability description (specificity, sensitivity) for each tool you have used to assess the outcome and associated factors.(World Health Organization (WHO) composite international diagnostic
interview (CIDI) assessment tool, Alcohol, Smoking, and Substance Involvement Screening Test (ASSIST), Depression, Anxiety, and Stress Scale - 21 (DASS-21) , Abuse Assessment Scale (AAS) and Oslo Social support Scale.

***Authors’ response: We have accepted the comment and we have tried to rewrite a reliability description (specificity, sensitivity) for each tools used in this study in the revised version of the manuscript. Thank you.***

3. It is better to describe some of the clinical variables ( Hypermesis gravidrum and gestational age information) that were taken from their medical card or chart)

***Authors’ response: Dear editor, thank you for the comment. We addressed the comment and described the variables that were taken from the medical charts at the end of data collection instruments section in the revised manuscript. Thank you.***

4. it is better to correct spelling errors ( at the regression table; medical was written as medical) and number alignment ( center , right and left , look at the regression table)

***Authors’ response: Dear editor, we have accepted the comment and corrected the spelling errors and number alignment. Thank you.***

5. Have you made statistical assumptions? If so, please describe: symptoms of suicidal ideation is also found in depressive symptoms of DASS.) Do you solve this collinearity? , state in detail on data analysis

***Authors’ response: Dear editor, thank you for the comment. We did the multi-collinearity and extreme outlier assumption checking by calculating Variance Inflation Factors (VIF) and residual respectively, including for suicidal component in the DASS with suicidal ideation. We have described and included this in the revised version of the manuscript. However, we didn’t check linearity assumption since no continuous independent variable was used in the data. Thank you.***

6. On your exclusion criteria, you did not mention any exclusion of those who have a previous history of mental illness. This might affect your outcome variable if you include how do you treat your data ( there are 3 in your data )

***Authors’ response: We have included those participants who have a previous of mental illness because they had minor mental health problem which is less likely to experience suicidal behavior and consequently less likely to impact the outcome variable. Therefore, we included them in order to avoid deliberate exclusion of the participants. Thank you.***

7. It is better to rewrite the title of the place of study. I mean, it misled a reader as it was conducted in all public hospitals found in eastern Ethiopia.

***Authors’ response: Dear editor, we have accepted the comment and rewrite the title as “Suicidal ideation and associated factors among pregnant women attending antenatal care at public hospitals of Harari regional state, eastern Ethiopia” as suggested. Thank you***

8. In the discussion , it is better to explain a difference other than data collection tool difference.

***Authors’ response: We have accepted the comment and we tried to describe the possible explanations for the discrepancy occurred between the current study and previous studies other than tool difference in the revised manuscript. Thank you.***

1. On the introduction section
   1.1. it is better to change the second reference 1 ( you cited two times )

***Authors’ response: We have accepted the comment and we changed it accordingly.***
2. Discussion
2.1. It is better to revise line number 315-325 , it is better to cite for each measurement of suicidal ideation e.g. EPDS ( cite here a study ) , (SBQ-R) ( cite here), ……..

***Authors’ response: We have accepted the comment and we cited the reference for each tool separately. You can see the revise manuscript.***
2.2. It is better to revise line number 330-341, it is better to add citation previous studies ( cite here) , Ethiopia (cite here , in study) and studies done in other countries ( cite here).

***We have accepted the comment and we cited accordingly. You can see the revise manuscript.***
2.3. It is better to revise line number 375-378 , It is not advisable to compare with USA studies , please find studies done in low income countries , Asian …..

***We have accepted the comment and we modified it.***
Finally it is better to revise spelling, grammar and vocabulary corrections on the whole section of a manuscript.

***We have tried to fix the grammar and spelling error in the revised manuscript.***

Reviewers' comments:

Reviewer #1: Dear authors,
Thank you for your contribution to the field. I have had the opportunity to review your manuscript and found it well-written. However, there are some points you need to address to improve the quality of your work, and I have mentioned them section by section as follows:
Title and Abstract
1. The title is interesting and it addresses the current public health problem, but you should rewrite it as “Suicidal ideation and its associated factor among pregnant women in the eastern part of Ethiopia: A cross-sectional study.”

***Authors’ response: Dear reviewer, we have accepted the comment and rewrite the title as “Suicidal ideation and associated factors among pregnant women attending antenatal care at public hospitals of Harari regional state, eastern Ethiopia: A cross-sectional study” as suggested by you and editor. Thank you.***

2. In the methods section of the abstract, you should incorporate the specific study area and study period.

***Authors’ response: We have accepted the comment and we included a specific study area and study period. Thank you.***

3.In the result section of the abstract, paraphrase the following sentence to make it easier for the reader: Experiencing intimate partner violence and stress (AOR= 3.46; at 95% CI= = 1.75–6.66) and (AOR = 2.88; at 95% CI = 1.11–7.36) were significantly associated with suicidal ideation among pregnant women. “

***Authors’ response: We have accepted the comment and paraphrase it accordingly in the revised manuscript. Thank you.***

4. Put keywords in ascending order

***Authors’ response: We have accepted the comment and we put the keywords in ascending order. Thank you.***

introduction  
5. Overall, the introduction is well-written and adheres to the systematic writing of the background (deductive approach), and it maintains coherence between paragraphs. However, try to improve the grammatical errors through proofreading.

***Authors’ response: Dear reviewer, thank you for the comment. We have tried to improve the grammatical error in the revised version of the manuscript.***

Methods  
The methods are clear, and the statistical approaches used in this study are appropriate. I have only three suggestions to improve the paper:
6. The sample size calculation formula does not need to be included in the calculation. The author just needs to convey what formula is used along with the margin of error set to get the required sample size.

***Authors’ response: We have accepted the comment and removed the formula as suggested. Thank you.***

7. In lines 102-107, you should specify your study area. In which hospital did you conduct your study? Let us know more about your study hospitals.

***Authors’ response: We have accepted the comment and specified the study area including the two public hospitals where the study was done. Thank you.***

8. In lines 120–122, why do you exclude those with hyperemesis gravidarum secondary to other medical conditions such as thyroid diseases and liver diseases after proven investigation? It needs further justification if thyroid diseases and liver diseases have a direct linkage with suicidal ideation; if it is so, don’t forget to cite your reference for your justification.

***Authors’ response: Thank you for the concern. One of the primary reasons for excluding individuals with hyperemesis gravidarum secondary to other medical conditions from the study was the complexity and heterogeneity of their medical condition. Thyroid diseases and liver diseases encompass a wide range of disorders, each with its own unique pathophysiology, clinical manifestations, and treatment approaches. Including individuals with hyperemesis gravidarum secondary to these diverse conditions in a study may introduce significant variability that could confound the results and make it challenging to draw meaningful conclusions. That is why we prefer to exclude them. Thank you.***

9. In line 127, why do you use a margin of error of 0.03 instead of 0.05?

***Authors’ response: We took proportion (P) of 13.3% from the previous study conducted in Jimma as a magnitude of suicidal ideation among pregnant women, which was resulting small sample size. Therefore, in order to increase the sample size, the margin error of 3% was taken. Thank you.***

10.Line 135-140 Why do you take average monthly attendants since your study period is 2 months? You calculated the k value in the wrong way. Please readjust it.

***Authors’ response: In order to get the average flow of attendants, we dividing the annual total number into months. However, we made an error on the calculating the K value, we supposed to take the two month period, and we readjusted it in the revised manuscript as “The interval size (k) is calculated using the following formula.*** $\boldsymbol{k=}\frac{\boldsymbol{N}}{\boldsymbol{n}}$ ***=2448/543 = 4.5 ≈4.So every four persons was selected from each hospital”. Thank you for your constructive comment.***

11. In Line 141, why do you select from the first three since your k value is 2?

***Authors’ response: We took the comment and corrected it in the revised version of the manuscript as “The first pregnant woman was selected from the first four by lottery method”. Thank you.***

12. In lines 143–146, you should provide the questionnaire that was used for your study in a supplementary file and cite it in the method section as a supplemental file.

***Authors’ response: We have accepted the comment and we have uploaded all the data underlying the findings described in the manuscript as supplementary information and we have cited it as supplemental file in data collection tool sub-section in the methods section of the revised the manuscript. Thank you.***

13. In Line 166 (IPV), you should provide both full words and abbreviations when you use it for the first time.

***Authors’ response: We have accepted and addressed the comment. Thank you.***

14. In lines 168–169, move the sentence and merge it under data quality control.

***Authors’ response: We have accepted and addressed the comment. Thank you.***
15. In data quality control, did you provide training for data collectors? If so, you should state it in your manuscript.

***Authors’ response: Yes we provide the training for data collectors regarding data collection methods, data collection tools, and how to handle ethical issues. We have stated this in the revised manuscript. Thank you.***

16. In Line 170-189, move your operational definitions above the data collection instruments and procedures.

***Authors’ response: We have accepted and addressed the comment. Thank you.***

17. Cite reference for the operational definitions of pregnant women, hyperemesis gravidarum, Suicidal ideation and sleep quality

***Authors’ response: We have accepted and addressed the comment. Thank you.***
 
Results
The results are relevant, but you should revise them based on the following comment to improve the manuscript.
18.Line 214, “{26.53” should be replaced by "26.53 years.”
19. Line 215, do you think the word most is suitable for 58.36%?
20. Line 217, delete “with the largest proportion."

***Authors’ response: We have accepted and addressed the comments from 18 to 20. Thank you.***

21. In Table 1, you should re-categorize the age by using the standard age category. If you have a study participant whose age is less than 18, you should specify it by adding the category <18 years, since it will provide us with good results.

***Authors’ response: Thank you for your concern. We have got only two participants who aged than 18 years and this small number makes us unable to compare them with other age category. That is why we have merged it as less than 20 years.***

22. It is difficult to assess the average monthly income for your study participants since either they are not civil servants or unless you did a wealth index.

***Authors’ response: We agree with your concern. Since we didn’t do the wealth index and less reliable to put it in income, we omit it from the revised manuscript. Thank you.***

23. Please try to change Table 4 into a pie chart format

***Authors’ response: We have accepted the comment and changed table 4 into pie chart. Thank you.***

24. Lines 260–262, rewrite each factor separately: “ The odds ratio of having suicidal ideation for a pregnant mother with depressive and anxiety symptoms were 2.79 and 3.37 compared to their counterparts (AOR = 2.79; (95% CI= = 1.49–5.23) and AOR = 3.37; (95% CI = 1.69–6.68), respectively.”

***Authors’ response: We have accepted the comment and we rewrite it as suggested. Thank you.***

25. In Table 5, age less than 20, how do you run the regression for cell value less than 5, which is "3"? It is a critical issue and must be addressed by re-categorizing the age value.

***Authors’ response: Thank you for your concern. The regression can run the cell value if it is not 0, but the total frequency of the specific category should be more than 5, which is 27 in this case. That is why we categorize it as mentioned in the manuscript. Thank you.***

26. In the Table 5 suicidal ideation column, put the suicidal value of yes before no for a better understanding

***Authors’ response: We have accepted and addressed the comment as suggested. Thank you.***

Discussion  
The discussions are explained well enough and based on the results. But to improve it, you should revise it based on the following comments:
27. In Line 282, try to justify how the tool difference is the possible reason for the variation. Try to justify by raising ideas about each tool and their possibility to increase or decrease their estimation of prevalence.  Please try to think beyond tool difference and inclusion criteria
28. Lines 290–292, try to justify how the tool difference is the possible reason for the variation. Try to justify by raising ideas about each tool and their possibility to increase or decrease their estimation of prevalence. Please try to think beyond tool difference and inclusion criteria

***Authors’ response: We have accepted the comment and tried to describe the possible explanations for the discrepancy occurred between the current study and previous studies other than tool difference in the revised manuscript. Additionally, we also elaborated how the tools difference affects the outcome (suicide). Thank you.***

29. Line 301, you should specify by stating the specific study area for “This is supported by studies (13–15).”

***Authors’ response: We have accepted and addressed the comment as suggested. Thank you.***

30. Line 301-303 is not directly linked to your justification. You should better delete it. “A cross-sectional study conducted in the United Kingdom reported that 52.1 percent of participants thought to terminate their pregnancy, and 4.9 percent of them terminated their pregnancy owing to hyperemesis gravidarum.”

***Authors’ response: We have accepted the comment and deleted the stated statement since it is not a relevant justification. Thank you.***

Strengths and Limitations of the study
31. Line 344, please clarify this sentence:evidence-based laboratory tests Which lab? To assess what?
32. In lines 344–345, it is good to state recall bias and social desirability bias as limitations, but you should better state your effort to reduce those biases in this section.
33.Line 344–346 is a non-sense paragraph; you are expected to paraphrase it “However, recall bias, social desirability, a cross-sectional study design that cannot allow establishing a temporal relationship between suicidal ideation, and significant associated factors were the limitations of the study.

***Authors’ response: For question 31 to 33, we have accepted the comments and we extensively rewrite the strength and limitations section in the revised version of the manuscript.***

Conclusion
34. Your conclusion is aligned with the implication of your study rather than the mere figure of the results and is also drawn from your main finding. I am okay with that.
35. In the recommendation section, why do you only stick with hyperemesis gravidarum since you have many findings?

***Authors’ response: We have accepted the comment and we added the recommendations for the remaining main findings. Thank you.***

Reviewer #2: 1.It will be more helpful to the readers if the setting where the study was conducted is mentioned in the abstract part.

***Authors’ response: We have accepted the comment and we included a specific setting were the study was conducted in the revised version of the manuscript. Thank you.***

2.It also makes the research more influential if the research owner gives full feedback on the findings. For example, unwanted pregnancy is a factor for SI, but there is nothing to what should be done

***Authors’ response: We have accepted the comment and we added the recommendations for the all main findings as suggested. Thank you.***

3.It would not be good to use one or two of the references that are out of date

***Authors’ response: We have accepted the comment and we have tried to use latest references. Thank you.***

4. The Overview of suicidal ideation among the study population is not organized well based on Global to local contexts. .

***Authors’ response: We have accepted the comment and re-arranged the introduction part as suggested. Thank you.***

5.author text document spacing should be double spacing according to the journal guide line

***Authors’ response: We have checked the journal guideline and made the adjustments to meet the journal requirements. Thank you.***
